# Obstructive Sleep Apnea: A Cluster Analysis at Time of Diagnosis

**DOI:** 10.1371/journal.pone.0157318

**Published:** 2016-06-17

**Authors:** Sébastien Bailly, Marie Destors, Yves Grillet, Philippe Richard, Bruno Stach, Isabelle Vivodtzev, Jean-Francois Timsit, Patrick Lévy, Renaud Tamisier, Jean-Louis Pépin, scientific council and investigators of the French national sleep apnea registry (OSFP)

**Affiliations:** 1 Univ. Grenoble Alpes, HP2; Inserm, U1042, Grenoble, France; 2 IAME UMR 1137 Inserm Université Paris Diderot, F-75018, Paris, France; 3 Grenoble- Alpes University Hospital, Laboratoire EFCR, Pôle THORAX et VAISSEAUX, Grenoble, France; 4 Pneumologist, Private Clinic, Valence, France; 5 Pneumologist, Private Clinic, St-Omer, France; 6 Pneumologist, Saint Michel Private Clinic, Valenciennes, France; 7 Medical and Infectious Diseases ICU - Paris Diderot University / Bichat Hospital, Paris, France; Charité University Medicine Berlin, GERMANY

## Abstract

**Background:**

The classification of obstructive sleep apnea is on the basis of sleep study criteria that may not adequately capture disease heterogeneity. Improved phenotyping may improve prognosis prediction and help select therapeutic strategies. *Objectives*: This study used cluster analysis to investigate the clinical clusters of obstructive sleep apnea.

**Methods:**

An ascending hierarchical cluster analysis was performed on baseline symptoms, physical examination, risk factor exposure and co-morbidities from 18,263 participants in the OSFP (French national registry of sleep apnea). The probability for criteria to be associated with a given cluster was assessed using odds ratios, determined by univariate logistic regression. *Results*: Six clusters were identified, in which patients varied considerably in age, sex, symptoms, obesity, co-morbidities and environmental risk factors. The main significant differences between clusters were minimally symptomatic versus sleepy obstructive sleep apnea patients, lean versus obese, and among obese patients different combinations of co-morbidities and environmental risk factors.

**Conclusions:**

Our cluster analysis identified six distinct clusters of obstructive sleep apnea. Our findings underscore the high degree of heterogeneity that exists within obstructive sleep apnea patients regarding clinical presentation, risk factors and consequences. This may help in both research and clinical practice for validating new prevention programs, in diagnosis and in decisions regarding therapeutic strategies.

## Introduction

Obstructive sleep apnea (OSA) is a major global health concern, causing considerable cardiovascular and metabolic morbidity and mortality [1]. Although mainly defined by the apnea + hypopnea index (AHI) [2], OSA is nowadays considered a complex, heterogeneous and multi-component condition. It is increasingly recognized that the presence of symptoms, mainly sleepiness but also comorbidities such as cardiovascular and metabolic disease, substantially contributes to prognosis [3].

Treatment for OSA is prescribed not only to normalize symptoms but in a significant percentage of less symptomatic patients it also represents a way of reducing cardiometabolic risk. However, continuous positive airway pressure (CPAP) the first line therapy of OSA fails to improve blood pressure and alter metabolic or inflammatory markers especially in minimally symptomatic obese OSA [4]. This emphasizes the need to offer a combination of multiple modalities of treatment including weight loss by lifestyle interventions or bariatric surgery, physical activity and new medications for reduction of cardiovascular risk specifically dedicated to OSA patients. The pre-requisite for implementing this “personalized OSA medicine” is to have identified the main OSA clusters.

There are few studies that have formally characterized the distinct combinations of symptoms and comorbidities in OSA patients [5, 6] as has been recently done for chronic diseases such as COPD or asthma [7]. We applied cluster analysis to examine the presence of clinically important patient subgroups within a well-characterized national prospective cohort of OSA patients.

## Material and Methods

### Study subjects and data source

We report a cluster analysis of data from a prospective national cohort, using the research database of the “Observatoire Sommeil de la Fédération de Pneumologie” (OSFP) (www.osfp.fr). The OSFP registry is a standardized web-based report, administered by the French Federation of Pulmonology. It contains anonymized longitudinal data from patients complaining of sleep disorders, recorded by respiratory physicians in private practice, general hospitals and university hospitals. Periodic quality control checks are performed to ensure up-to-standard data recording. Ethical committee approval for setting up the database was obtained from “Le Comité consultatif sur le traitement de l’information en matière de recherche en santé” (C.C.T.I.R.S n° 09.521) and authorization from the “Commission Nationale Informatique et Liberté” (C.N.I.L), the French information technology and personal data protection authority. The OSFP Independent Scientific Advisory Committee approved data use for this study. All patients included in the database gave written informed consent.

### Assessments

Patients, over 18 years of age, who had a diagnosis of obstructive sleep apnea syndrome (OSAS) (Apnea/Hypopnea Index (AHI) > 15 events/hour or Oxygen Desaturation Index (ODI) > 15 events/hour) were included in our data analysis.

The following data, collected at the first medical visit, are recorded: demographic characteristics, scores (Epworth Sleepiness Scale (ESS), Pichot fatigue scale and the Pichot depression scale), subjective sleep duration, OSAS symptoms, blood pressure, waist circumference and co-morbidities (cardio-vascular, metabolic and respiratory). We also recorded available environmental risk factors such as smoking and alcohol as well as sedentarity.

### Statistical analysis

Before cluster analysis, data with less than 5% of missing values were imputed using the median for quantitative variables (age, height, weight, AHI), and using multiple imputation for qualitative variables. A multiple correspondence analysis (MCA) was performed using clinical information: clinical symptoms, co-morbidities, and patients’ anthropometric characteristics (age, sex, Body Mass Index (BMI)). The number of dimensions for MCA was identified using Kaiser criteria and the Scree test [8]. Individual coordinates obtained with MCA were introduced in a disjoint cluster analysis to identify and exclude outliers (clusters with less than 20 individuals). A final ascending hierarchical clustering analysis was performed using the individual coordinates. The ascending hierarchical clustering begins with each patient as a separate cluster and merges them into successively larger clusters. Ward’s method was considered to minimize the within-cluster sum of squares. The final number of clusters was defined on the basis of the cubic clustering criterion, the Pseudo-t squared and the Pseudo F Statistic. Variables were described separately for each cluster by the use of median and interquartile range (IQR) for quantitative variables and frequency and percent for qualitative variables. Comparisons between clusters were performed using ANOVA or Kruskall-Wallis test for quantitative variables and Chi square test or Fisher exact test for qualitative variables. The probability for criteria to belong at one cluster was assessed using odds ratios, determined by a univariate logistic regression and probability within each cluster was shown using radar charts. Statistical analysis was performed using SAS v9.3 (SAS Inc, NC, USA).

## Results

### Study flow

Among patients registered in the OSFP database, 18,263 patients were diagnosed as having OSAS and included in the cluster analysis after exclusion of 426 (2%) patients under 18 years old or with incomplete or aberrant data.

### Patient characteristics

The median age of our OSA patients was 59 (Inter quartile range (IQR): 50–67) years with 72.8% of males. They had a median BMI of 31 (27–36) kg/m^2^ (Table 1).

**Table 1:** Patients characteristics of the entire cohort and by clusters: Anthropometric and demographic characteristics. Values in Numbers (%) or median [IQR]. Body Mass Index (BMI). Cluster 1: the young symptomatic. Cluster 2: the old obese. Cluster 3: the multi-disease (MD) old obese. Cluster 4: the young snorers. Cluster 5: the drowsy obese. Cluster 6: the MD obese symptomatic.

	All clusters	Cluster 1	Cluster 2	Cluster 3	Cluster 4	Cluster 5	Cluster 6
	N = 18,263	N = 1,823	N = 4,200	N = 3,363	N = 2,715	N = 3,511	N = 2,642
Age (years)	59 [50; 67]	48 [41;55]	63 [56;71]	66 [60;74]	49 [40;57]	56 [48;63]	60 [54;66]
Gender (male)	13,465 (73.8)	1,427 (78.3)	3,138 (74.7)	2,349 (69.8)	2,209 (81.4)	2,432 (69.3)	1,910 (72.3)
BMI (kg/m^2^)	31 [27;36]	29 [26;35]	31 [27;35]	33 [29;38]	28 [25;33]	31 [27;36]	33 [29;37]
Waist circumference (cm)	109 [100;120]	104 [96;116]	108 [100;119]	115 [106;124]	100 [92;111]	108 [99;118]	113 [104;122]
Sedentary	3071 (16.8)	233 (12.8)	272 (6.5)	1021 (30.4)	90 (3.3)	538 (15.3)	917 (34.7)
Current smoker	2838 (15.5)	562 (30.8)	357 (8.5)	207 (6.2)	721 (26.5)	632 (18)	360 (13.6)
Former smoker	5366 (29.4)	338 (18.5)	1156 (27.5)	1570 (46.7)	324 (11.9)	912 (26)	1068 (40.4)
Systolic Blood pressure (mmHg)	130 [125;140]	130 [120;140]	130 [130;140]	140 [130;147]	130 [120;140]	130 [124;140]	140 [130;150]
Diastolic Blood pressure (mmHg)	80 [70;86]	80 [70;86]	80 [70;84]	80 [70;85]	80 [70;80]	80 [70;88]	80 [70;90]

Hypertension was the most frequently observed co-morbidity (46.4%). Dyslipidemia was present in 30.4% and diabetes in 14.7% of the included patients (Table 2).

**Table 2:** Patients characteristics of the entire cohort and by clusters: Co-morbidities. Values in Numbers (%).

	All clusters	Cluster 1	Cluster 2	Cluster 3	Cluster 4	Cluster 5	Cluster 6
	N = 18,263	N = 1,823	N = 4,200	N = 3,363	N = 2,715	N = 3,511	N = 2,642
**Cardiovascular and metabolic co-morbidities**							
Coronary heart disease	1402 (7.7)	19 (1)	295 (7)	642 (19.1)	17 (0.6)	101 (2.9)	328 (12.4)
Arrhythmias	1562 (8.6)	28 (1.5)	343 (8.2)	689 (20.5)	22 (0.8)	139 (4)	341 (12.9)
Stroke	594 (3.3)	13 (0.7)	141 (3.4)	244 (7.3)	10 (0.4)	63 (1.8)	124 (4.7)
Heart failure	538 (2.9)	11 (0.6)	116 (2.8)	250 (7.4)	10 (0.4)	54 (1.5)	99 (3.7)
Hypertension	8462 (46.4)	254 (13.9)	2050 (48.8)	2857 (84.9)	115 (4.2)	1203 (34.3)	1988 (75.1)
Diabetes	2683 (14.7)	44 (2.4)	408 (9.7)	1306 (38.8)	7 (0.3)	221 (6.3)	697 (26.4)
Dyslipidemia	5552 (30.4)	195 (10.7)	922 (22)	2090 (62.1)	104 (3.8)	834 (23.8)	1407 (53.3)
**Other co-morbidities**							
Respiratory co-morbidities *	1171 (6.4)	56 (3.1)	229 (5.5)	388 (11.5)	56 (2.1)	178 (5.1)	264 (10)
Depression	2573 (14.1)	229 (12.6)	325 (7.7)	604 (18)	142 (5.2)	578 (16.5)	698 (26.4)

* COPD, Asthma and chronic respiratory failure.

Cluster 1: the young symptomatic. Cluster 2: the old obese. Cluster 3: the multi-disease (MD) old obese. Cluster 4: the young snorers. Cluster 5: the drowsy obese. Cluster 6: the MD obese symptomatic

The three most common OSA symptoms observed were: snoring (92.4%), self-reported daytime sleepiness (79.2%) with a median ESS of 10 (6–14) and morning fatigue (69.8%). The majority of OSAS patients exhibited moderate to severe disease with a median AHI of 35 (25.5–50.8) events/hour and a median ODI of 27 (16–44.5) events/hour (Table 3).

**Table 3:** Patients characteristics of the entire cohort and by clusters: Sleep characteristics, OSAS symptoms and functional scales. Values in Numbers (%) or median [IQR]. Obstructive Sleep Apnea Syndrome (OSAS), Apnea/Hypopnea Index (AHI), Oxygen Desaturation Index (ODI). Cluster 1: the young symptomatic. Cluster 2: the old obese. Cluster 3: the multi-disease (MD) old obese. Cluster 4: the young snorers. Cluster 5: the drowsy obese. Cluster 6: the MD obese symptomatic.

	All cluster	Cluster 1	Cluster 2	Cluster 3	Cluster 4	Cluster 5	Cluster 6
	N = 18,263	N = 1,823	N = 4,200	N = 3,363	N = 2,715	N = 3,511	N = 2,642
**Sleep characteristics**							
Self-reported sleep duration (hours)	7 [6; 8]	7 [6;8]	7.5 [6.5;8]	7.5 [6;8]	7 [6;8]	7 [6;8]	7 [6;8]
Short sleeper (<6h)	1465 (8)	168 (13.2)	206 (11)	284 (12.1)	175 (11.8)	342 (13.6)	290 (14.1)
Intermediate sleeper (6h-9h)	8558 (46.9)	970 (76.1)	1429 (76.3)	1662 (71.1)	1190 (80.5)	1860 (73.8)	1447 (70.4)
Very long sleeper (>9h)	1516 (8.3)	136 (10.7)	239 (12.8)	393 (16.8)	113 (7.6)	317 (12.6)	318 (15.5)
AHI (/h)	35 [26; 51]	31.6 [22;47]	34 [26;47.4]	40 [30;55]	31 [22;42]	34 [25;50]	39 [29;58]
ODI (/h)	27 [16; 45]	23 [13;40.9]	26 [15.9;41]	33 [21;50]	21 [11.8;35]	25 [14.2;42]	31 [18;51]
Time spent with nocturnal SaO2 below 90% (%)	34 [9; 104]	20 [5;72]	32 [9;91]	59 [18;148]	15 [4;50]	29 [8;89]	49 [15;130]
**OSAS symptoms**							
Snoring	16955 (92.4)	1818 (99.7)	3314 (78.8)	3226 (95.9)	2484 (91.5)	3434 (97.8)	2614 (98.8)
Self-declared daytime sleepiness	14451 (79.2)	1812 (99.4)	2261 (53.8)	2755 (81.9)	1856 (68.3)	3197 (91.1)	2577 (97.4)
Morning fatigue	12740 (69.8)	1770 (97.1)	1618 (38.5)	2293 (68.2)	1612 (59.4)	2975 (84.7)	2480 (93.7)
Nocturia	10647 (58.3)	1060 (58.1)	1670 (39.7)	2695 (80.1)	672 (24.7)	2361 (67.2)	2194 (82.9)
Headaches	6578 (36)	1567 (86)	446 (10.6)	632 (18.8)	667 (24.6)	1544 (44)	1726 (65.2)
Near miss accident	1389 (7.6)	348 (19.1)	77 (1.8)	133 (4)	158 (5.8)	305 (8.7)	368 (13.9)
**Functional scales**							
Pichot Scale	13 [6; 20]	16 [9;22]	7.5 [3;14]	12 [6;19]	10 [4;17]	14 [8;20]	16 [10;22]
Depression scale	3 [1; 7]	4 [1;7]	2 [0;5]	3 [1;7]	2 [0;5]	4 [1;7]	5 [2;8]
Epworth scale	10 [6; 14]	12 [8;15]	8 [5;12]	9 [6;13]	10 [6;14]	11 [7;14]	11 [7;14]

### Cluster analysis

**Fig 1:**
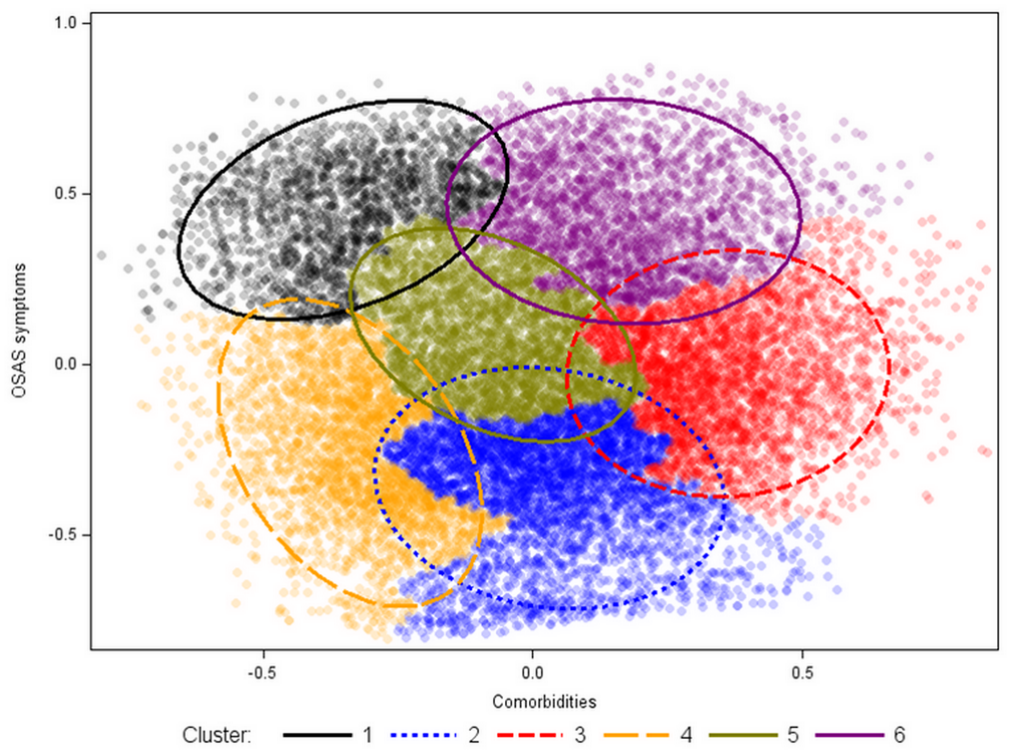
Representation of six clusters after ascending hierarchical clustering analysis. Axes correspond to individual coordinates for the two main dimensions of the multiple correspondence analysis. Cluster 1: the young symptomatic. Cluster 2: the old obese. Cluster 3: the multi-disease (MD) old obese. Cluster 4: the young snorers. Cluster 5: the drowsy obese. Cluster 6: the MD obese symptomatic.

The number of clusters identified after analysis was 6 (Fig 1).

#### Cluster 1 (1,823 patients, 10%): Young, overweight, symptomatic OSAS without co-morbidities: the young symptomatic

This cluster was representative of young (median age 48 (41–55)) and overweight (median BMI of 29 (26–35) kg/m^2^) OSA patients, with few or no co-morbidities compared to other clusters. The proportion of patients with major OSAS symptoms (except nocturia) was highest in Cluster 1. They had a median ESS of 12 (8–15) and 19.1% of them have already experienced near-miss road accidents.

#### Cluster 2 (4,200 patients, 23%): Elderly minimally symptomatic obese OSA with few co-morbidities: the older obese

Cluster 2 included the most patients. In contrast to Cluster 1, the lowest proportion of individuals with OSAS symptoms was in Cluster 2 (median ESS of 8 (5–12)). They were in the median range for BMI and OSA severity and exhibited few co-morbidities.

#### Cluster 3 (3,363 patients, 18.4%): Elderly minimally symptomatic multimorbid OSA: the multi-disease (MD) old obese

This cluster was composed of the oldest patients (median age 66 (40–74)). In the cohort, these patients were among the most obese (median BMI of 33 (29–38) kg/m^2^) and the most severe in terms of OSAS (median AHI of 40 (30–55) events/h and median ODI of 33 (21–50) events/h) but with few or no symptoms. Cluster 3 had the highest proportion of patients with co-morbidities (cardiovascular, metabolic, respiratory and others).

#### Cluster 4 (2,715 patients, 14.9%): Young, overweight, minimally symptomatic OSAS without co-morbidities: the young snorers

Patients were similar to Cluster 1, young (median age 49 (40–57)) and the less obese (median BMI of 28 (25–33) kg/m^2^), but apart from snoring, they had few or no OSAS symptoms. Cluster 4 had the smallest median AHI (31 (22–42) events/h) and ODI (21 (11.8–35) events/h) and the lowest proportion of patients with co-morbidities.

#### Cluster 5 (3,511 patients, 19.2%): Middle age, with few symptoms of OSA and few co-morbidities: the drowsy obese

This cluster was considered as an intermediate cluster with a median age of 56 (48–63), a median BMI of 31 (27–36) kg/m^2^, few OSAS symptoms and few co-morbidities. However, they experience daytime drowsiness (ESS 11).

#### Cluster 6 (2,642 patients, 14.5%): Middle age, symptomatic multimorbid OSA with particularly poor lifestyle habits: the MD obese symptomatic

Patients were similar to Cluster 3 in terms of obesity, severity of OSAS and to a lesser extent, co-morbidities. However, they were younger than Cluster 3 (median age 60 (54–66)) and had more OSAS symptoms than Cluster 2 to 5. They more frequently complained of nocturia. Moreover, these patients were the most sedentary, the biggest alcohol consumers and suffered from depression.

The main features of the different clusters are summarized in Table 4 with the odds ratios for comparisons of probabilities between clusters.

**Table 4:** Summary of main features of the different clusters. In bracket: median values for variables.

	Age (years)	BMI (kg/m^2^)	AHI (/h)	ODI (/h)	Epworth scale	Co-morbitidies	OSAS symptoms
Cluster 1	**Youngest (48)**	Low* (29)	Low (31.6)	Low (23)	**High** (12)	Few or no	**Many**
Cluster 2	**Oldest** (63)	Median (31)	Median (34)	Median (26)	Low (8)	Few or no	Few or no
Cluster 3	**Oldest** (66)	**Obese** (33)	**High** (40)	**High** (33)	Low (9)	**Many**	Few or no
Cluster 4	Young (49)	Low (28)	Low (31)	Low (21)	Median (10)	Few or no	Few or no
Cluster 5	Middle age (56)	Median (31)	Median (34)	Median (25)	**High** (11)	Few or no	Few or no
Cluster 6	Middle age (60)	**Obese** (33)	**High** (39)	**High** (31)	**High** (11)	**Many**	**Many**

*Low, median and high were defined by inter-cluster comparison.

Body Mass Index (BMI), Apnea/Hypopnea Index (AHI), Oxygen Desaturation Index (ODI), Obstructive Sleep Apnea Syndrome (OSAS). Cluster 1: the young symptomatic. Cluster 2: the old obese. Cluster 3: the multi-disease (MD) old obese. Cluster 4: the young snorers. Cluster 5: the drowsy obese. Cluster 6: the MD obese symptomatic

**Fig 2:**
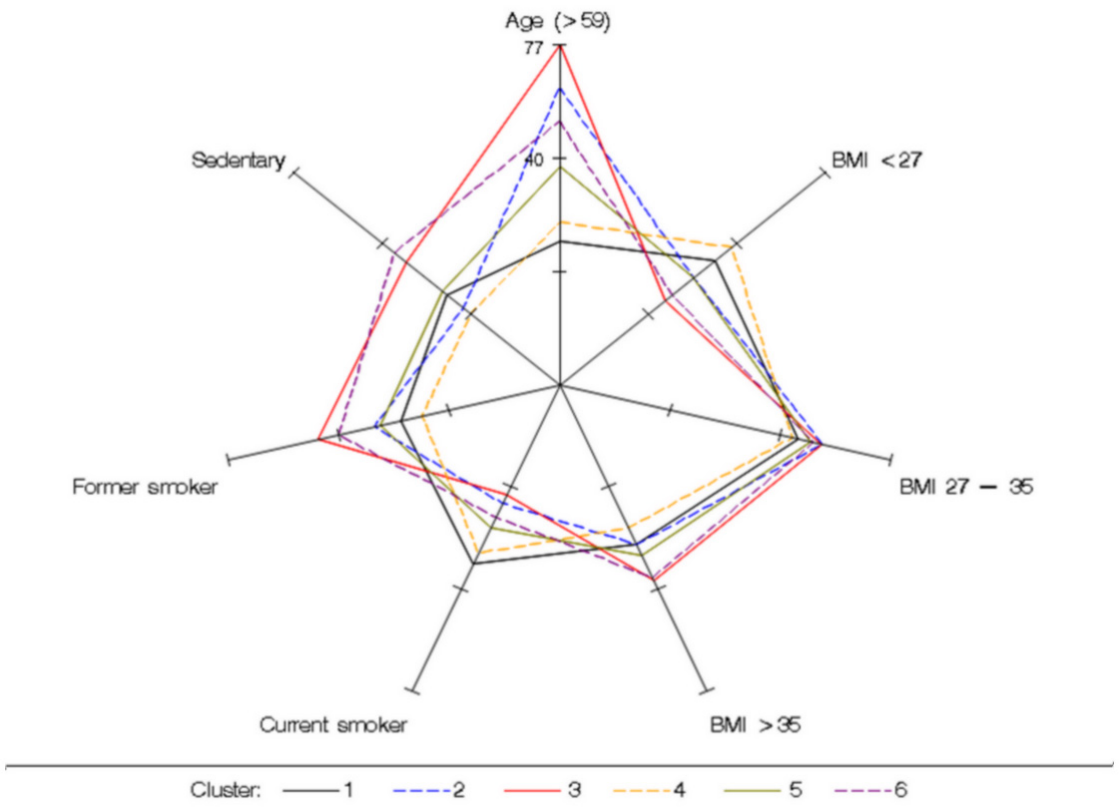
Conditional probabilities of BMI, age and risk factors to highlight the major differences among clusters. Cluster 1: the young symptomatic. Cluster 2: the old obese. Cluster 3: the multi-disease (MD) old obese. Cluster 4: the young snorers. Cluster 5: the drowsy obese. Cluster 6: the MD obese symptomatic

**Fig 3:**
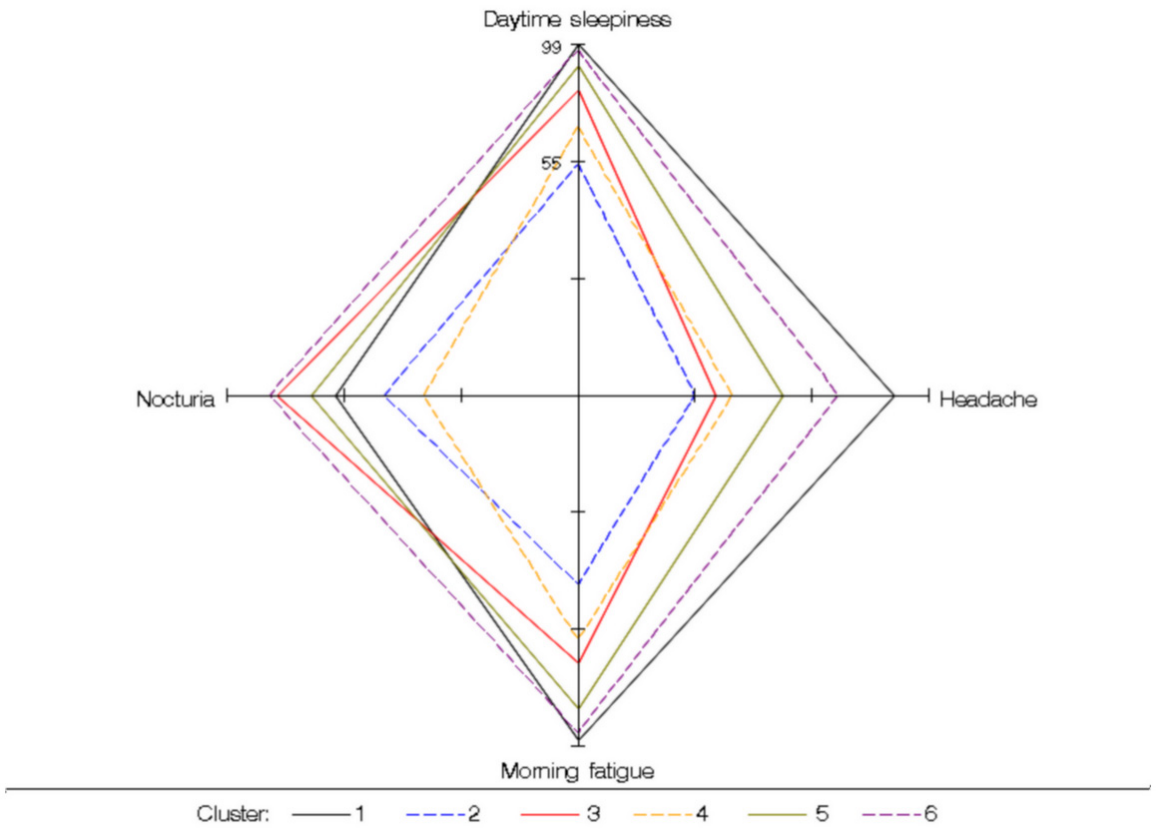
Conditional probabilities of symptoms to highlight the major differences among clusters. Cluster 1: the young symptomatic. Cluster 2: the old obese. Cluster 3: the multi-disease (MD) old obese. Cluster 4: the young snorers. Cluster 5: the drowsy obese. Cluster 6: the MD obese symptomatic

**Fig 4:**
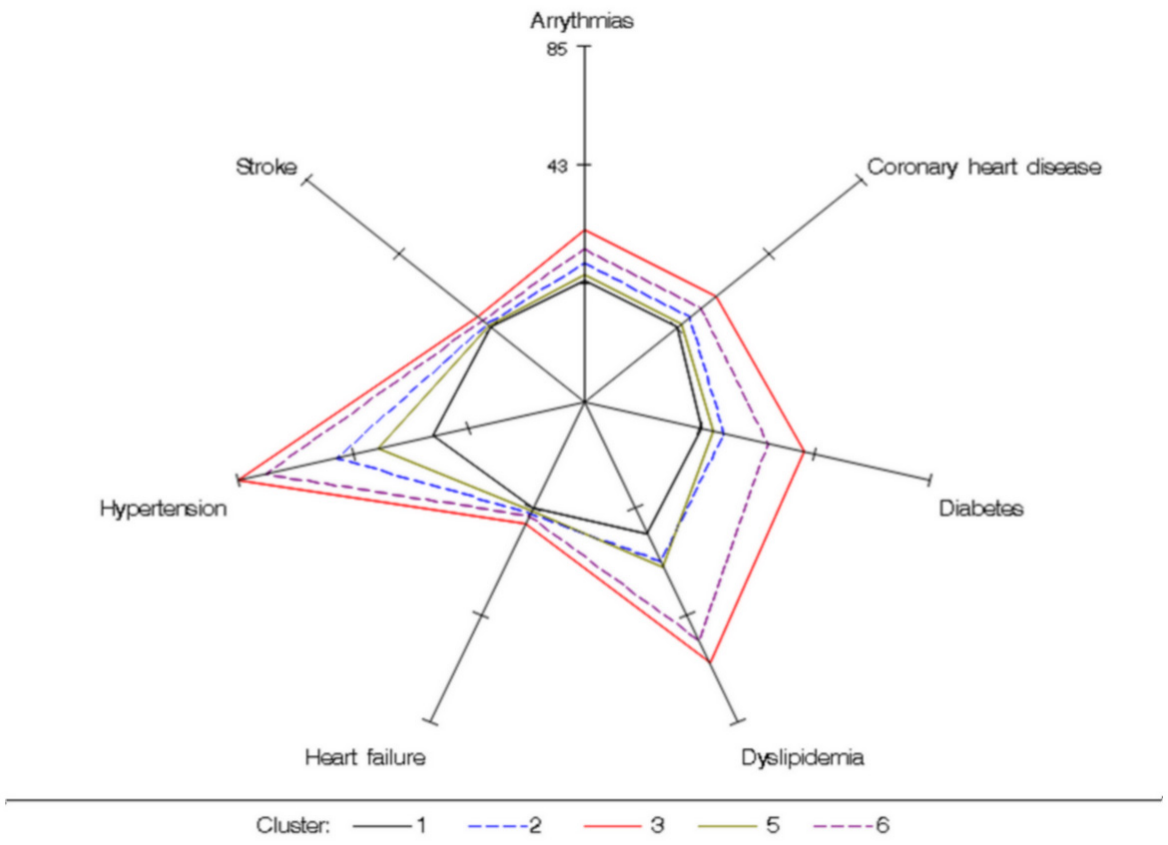
Conditional probabilities of co-morbidities to highlight the major differences among clusters. Cluster 4 was not represented in this figure because the probability to have cardiovascular and metabolic co-morbidities was near 0. Cluster 1: the young symptomatic. Cluster 2: the old obese. Cluster 3: the multi-disease (MD) old obese. Cluster 5: the drowsy obese. Cluster 6: the MD obese symptomatic

Figs 2 to 4 show the probabilities of having certain demographic characteristics, OSAS symptoms, risk factors and co-morbidities across clusters.

There were two groups of patients with significant OSAS symptoms (Fig 3): *the young symptomatic* (Cluster 1), young and thin patients with mild OSAS and few or no co-morbidities, and *the MD obese symptomatic* (Cluster 6), middle-aged and obese patients with severe OSAS and significant co-morbidities (cardiovascular, metabolic and respiratory). Nocturia was particularly frequent in *the MD obese symptomatic*.

*The MD old obese* (Cluster 3) and *the MD obese symptomatic* had the highest proportion of patients with significant cardiovascular and metabolic co-morbidities (Fig 4) and with severe OSAS (Table in S1 Table). The differences between these two clusters were age, OSAS symptom severity and lifestyle. The patients in *the MD obese symptomatic* were younger, more symptomatic and more sedentary.

## Discussion

Our study is the largest cluster analysis to date conducted in the field of mostly obese patients with obstructive sleep apnea syndrome. Previous studies addressing OSA clusters were limited by their small sample size and relatively low number of variables included in the analysis. In our large group of more than 18,000 OSA patients from a prospective national registry, patients within each cluster varied considerably in age, BMI, symptoms, co-morbidities, and risk exposures. Our data collection was multisite both from large academic sites and smaller private clinical practices implying that our results have high external validity and are highly generalizable. Our findings underscore the significant heterogeneity that exists between OSA patients at time of diagnosis.

Our study is a cross-sectional analysis using data collected at the time of diagnosis in patients referred to respiratory physicians. The same methodology has been recently used in a significantly smaller sample for the analysis of baseline data from the Icelandic Sleep Apnoea Cohort (822 patients with newly diagnosed moderate-to-severe OSA) [5]. Our data do not reflect the early expression and the natural history of all clusters in OSA. When we compare our clinical cohort to the Sao Paulo Epidemiologic Sleep Study [9] conducted in the general population, there are expected differences regarding age, BMI and co-morbidities. Clearly our data reflect a precise clinical context, with a snapshot at the moment at which the clinician has to propose therapeutic options. Using a huge sample based on the diagnostic code for OSA in health insurances databases, Mohklesi and colleagues have confirmed the high burden of co-morbidities in OSA patients [10]. These data also suggest that the clusters certainly evolve across lifespans as the divergence between OSA and controls was much more pronounced after the sixth decade of life for most cardiovascular diseases, while depression exhibited an opposite trend [10].

Two out of the six clusters demonstrated a significant combination of OSAS symptoms including sleepiness (24.5% of the patients). These two clusters were associated with the highest rates of near-miss accidents attributed to sleepiness. *The young symptomatic* (Cluster 1) was characterised by young overweight patients with mild OSAS and few or no co-morbidities. In this cluster the therapeutic strategy should be symptom-driven and both CPAP and alternatives to CPAP need to be considered. Mild OSA patients are generally believed to be poor CPAP compliers but symptomatic patients from *the young symptomatic* certainly had a greater likelihood to be CPAP adherent [11]. On the other hand, as they suffer from mild OSA without morbid obesity this cluster of individuals should also be anticipated to demonstrate a good response to non-CPAP therapies such as oral appliances and positional therapy [12]. The members of *the young symptomatic* and *the young snorers* (Cluster 4) were comparable in age with similar severity of OSA. Despite the same severity and duration of exposure to intermittent hypoxia, symptoms and neurocognitive and cardiovascular consequences were completely different in these two subtypes of OSA. This suggests the sleepy patient cluster is a subgroup with particular (genetically determined?) brain susceptibility to hypoxic exposure [13, 14]. This modulation of susceptibility to intermittent hypoxia has also been reported for cardio-metabolic consequences. This might explain the distinct natural course of different OSA clusters, and potentially, differential responses to CPAP [15]. In the two older age clusters, one experienced more severe intermittent hypoxia which was associated with many co-morbidities, as expected. The elderly members of *the old obese* (Cluster 2) suffered from mild to moderate OSA and were free of co-morbidities. In the elderly, mild-moderate OSA may confer cardio-protection by activating mechanisms of ischemic preconditioning [16, 17]. In animal models mimicking OSA exposure, mild-moderate intermittent hypoxia (IH) induces activation of cardio–protective mechanisms, whereas exposure to severe IH leads to damage of target organs [18]. The major challenge in personalized medicine for OSA patients is to identify clusters and biomarkers that define disease in terms of combined clinical, pathophysiological and biological abnormalities allowing therapeutic responses to be anticipated [19].

Apart from *the young symptomatic* (Cluster 1), the other “symptomatic” cluster (*the MD obese symptomatic*, Cluster 6) was characterized by middle-aged and obese patients with severe OSAS and significant co-morbidities (cardiovascular, metabolic and respiratory). Nocturia was particularly observed in *the MD obese symptomatic*. Nocturia is a common bothersome complaint in Obstructive Sleep Apnea (OSA) patients and is linked, in the general population, to an increased risk of hypertension and mortality. We recently demonstrated that nocturia is also a strong independent predictor of prevalent hypertension in OSA patients [20]. The members of *the MD obese symptomatic* exhibited severe intermittent hypoxia, the landmark of OSA, with certainly associated sympathetic over-activity and huge variations in intra-thoracic pressures against upper airway collapse that together underlie an increased secretion of natriuretic hormones and thereby nocturia [21].

A majority of clusters showed low rates of symptoms. These patients have probably been referred as they were “at-risk” of OSA. Obstructive sleep apnea is now considered as a risk factor for hypertension, stroke, coronary artery disease, congestive heart failure and arrhythmias [1]. Patients with cardiovascular disorders are frequently non-sleepy and less symptomatic [22, 23].

Another strength and novelty of our data-set was to provide information regarding risk exposures across the clusters (Fig 2). The middle age members of *the MD obese symptomatic* (Cluster 6) were symptomatic obese multimorbid OSA patients with particularly poor lifestyle habits. This particular cluster is clearly indicated for multimodal therapies including weight loss and exercise. In a comparable population, Chirinos et al. [24] have assessed the incremental effect of combined intervention including CPAP and weight loss over each alone. CPAP therapy alone did not have a significant effect on inflammation, insulin sensitivity, or dyslipidemia, even among participants who adhered to the therapy. However, there was a larger reduction in blood pressure (BP) in the combined intervention group that was compliant to CPAP than in either the weight loss group or the CPAP group. This seems also true when combining pharmacological treatment for systemic hypertension with CPAP, resulting in a better control of BP, particularly at night [25]. A recent study has demonstrated that aggressive multimodal risk factor management including OSA treatment improved the long-term success of atrial fibrillation ablation [26]. This underscores the importance of including OSA when introducing multimodal therapies directed at the primary promoters of the cardio-metabolic diseases. Sleep disorders should then be accepted as potential contributors to disparities in cardiovascular health [27].

Despite the many unique aspects of the manuscript (e.g., cluster analysis, large sample size), the present work has some limitations. First, the cut-off for AHI was >15 events per hour and this could have excluded a significant number of patients with AHI scores between 5 and14; however our study was designed to mainly focus on the moderate to severe OSA population who require treatment intervention. Second, the cluster analysis was based on the diagnosis visit data. It should be noted that OSA and individual responses are not static and evolve over time; thus further studies are needed to evaluate the time course of fluctuations in these clusters. Third, obesity on its own has a significant independent role on a number of cardio-vascular comorbidities and treatment of obesity may have a greater impact than OSA treatment on these comorbidities. Moreover, although a decrease of 10 to 15% of BMI may dramatically decrease upper airway flow obstruction [28], the same change in obesity is not sufficient to reduce the impact of obesity on cardio metabolic consequences. Therefore, it is interesting and essential to separate the effects of obesity per se from those related to OSA. However, our study was not designed to respond to this specific needful question.

## Conclusions

Our study that included 18,000 unselected OSA patients in a cluster analysis exploiting a large set of variables collected at time of diagnosis. We identified 6 distinct clusters of obstructive sleep apnea. Our findings underscore the high degree of heterogeneity that exists within obstructive sleep apnea patients regarding clinical presentation, risk factors and consequences. Obviously, these clusters need to be evaluated to determine whether thy have long-term prognostic implications.

## Supporting Information

S1 Table Odds ratios for comparisons of probabilities between clusters. (DOCX)
