# Supplementary material for: Obstructive Sleep Apnea: A Cluster Analysis at Time of Diagnosis
Source: PLoS One. 2016 Jun 17;11(6):e0157318. doi: 10.1371/journal.pone.0157318 (PMC4912165; doi:10.1371/journal.pone.0157318)
Supplement: S1 Table — (DOCX) [file pone.0157318.s001.docx]

**S1 Table. Odds ratios for comparisons of probabilities between clusters.**

|  |  | **Clusters** | | | | |
| --- | --- | --- | --- | --- | --- | --- |
|  |  | **1 vs 5** | **2 vs 5** | **3 vs 5** | **4 vs 5** | **6 vs 5** |
| **Anthropometric characteristics** | |  |  |  |  |  |
|  | Age (>59 years) | 0.3 [0.2 ; 0.3] | 2.9 [2.6 ; 3.2] | 5.6 [5 ; 6.2] | 0.4 [0.4 ; 0.4] | 1.9 [1.7 ; 2.1] |
|  | Sex (male) | 1.6 [1.4 ; 1.8] | 1.3 [1.2 ; 1.4] | 1 [0.9 ; 1.1] | 1.9 [1.7 ; 2.2] | 1.2 [1 ; 1.3] |
|  | BMI <27 | 1.5 [1.3 ; 1.7] | 1 [0.9 ; 1.1] | 0.4 [0.3 ; 0.4] | 2.2 [1.9 ; 2.4] | 0.5 [0.4 ; 0.6] |
|  | BMI 27 - 35 | 0.9 [0.8 ; 1] | 1.2 [1.1 ; 1.3] | 1.2 [1.1 ; 1.3] | 0.8 [0.7 ; 0.9] | 1.1 [1 ; 1.2] |
|  | BMI >35 | 0.8 [0.7 ; 0.9] | 0.8 [0.8 ; 0.9] | 1.5 [1.4 ; 1.7] | 0.6 [0.5 ; 0.6] | 1.5 [1.3 ; 1.6] |
|  | Waist circumference (>109) | 0.9 [0.8 ; 1] | 0.6 [0.5 ; 0.7] | 1.5 [1.4 ; 1.7] | 0.4 [0.3 ; 0.4] | 2 [1.8 ; 2.2] |
| **Risk factors and environmental exposures** | |  |  |  |  |  |
|  | Sedentary | 0.8 [0.7 ; 1] | 0.4 [0.3 ; 0.4] | 2.4 [2.1 ; 2.7] | 0.2 [0.2 ; 0.2] | 2.9 [2.6 ; 3.3] |
|  | Current smoker | 2 [1.8 ; 2.3] | 0.4 [0.4 ; 0.5] | 0.3 [0.3 ; 0.4] | 1.6 [1.5 ; 1.9] | 0.7 [0.6 ; 0.8] |
|  | Former smoker | 0.6 [0.6 ; 0.7] | 1.1 [1 ; 1.2] | 2.5 [2.3 ; 2.8] | 0.4 [0.3 ; 0.4] | 1.9 [1.7 ; 2.2] |
|  | Alcohol consumption | 0.9 [0.7 ; 1.2] | 0.6 [0.5 ; 0.8] | 1.3 [1.1 ; 1.7] | 0.5 [0.4 ; 0.7] | 1.8 [1.4 ; 2.2] |
| **Sleep characteristics** | |  |  |  |  |  |
|  | AHI 15 – 30 | 1.4 [1.2 ; 1.5] | 1 [0.9 ; 1.1] | 0.6 [0.6 ; 0.7] | 1.4 [1.3 ; 1.6] | 0.7 [0.7 ; 0.8] |
|  | AHI>30 | 0.7 [0.6 ; 0.8] | 1 [0.9 ; 1.1] | 1.6 [1.5 ; 1.8] | 0.7 [0.6 ; 0.8] | 1.4 [1.3 ; 1.6] |
|  | ODI 15 - 30 | 1 [0.9 ; 1.2] | 0.8 [0.7 ; 0.9] | 0.9 [0.8 ; 1] | 0.9 [0.8 ; 1] | 1 [0.9 ; 1.1] |
|  | ODI > 30 | 0.9 [0.8 ; 1.1] | 0.7 [0.7 ; 0.8] | 1.5 [1.4 ; 1.7] | 0.6 [0.5 ; 0.6] | 1.6 [1.5 ; 1.8] |
|  | Time spent with nocturnal SaO2 below 90% (>34) | 0.7 [0.6 ; 0.8] | 0.8 [0.7 ; 0.8] | 1.8 [1.6 ; 2] | 0.5 [0.4 ; 0.5] | 1.7 [1.5 ; 1.9] |
|  | Short sleeper (<6h) | 0.9 [0.8 ; 1.1] | 0.5 [0.4 ; 0.6] | 0.9 [0.7 ; 1] | 0.6 [0.5 ; 0.8] | 1.1 [1 ; 1.3] |
|  | Intermediate sleep duration (6-9h) | 1 [0.9 ; 1.1] | 0.5 [0.4 ; 0.5] | 0.9 [0.8 ; 1] | 0.7 [0.6 ; 0.8] | 1.1 [1 ; 1.2] |
|  | Very long sleeper (>9h) | 0.8 [0.7 ; 1] | 0.6 [0.5 ; 0.7] | 1.3 [1.1 ; 1.6] | 0.4 [0.4 ; 0.5] | 1.4 [1.2 ; 1.6] |
| **Epworth sleepiness scale** |  |  |  |  |  |  |
|  | Epworth scale <8 | 0.7 [0.6 ; 0.8] | 1.3 [1.2 ; 1.5] | 1.5 [1.3 ; 1.6] | 1 [0.9 ; 1.1] | 1 [0.9 ; 1.1] |
|  | Epworth scale 8 - 12 | 1.1 [1 ; 1.2] | 0.7 [0.6 ; 0.7] | 0.9 [0.8 ; 1] | 0.9 [0.8 ; 1] | 1.1 [1 ; 1.2] |
|  | Epworth scale >12 | 1.5 [1.3 ; 1.7] | 0.4 [0.4 ; 0.5] | 0.6 [0.5 ; 0.7] | 0.7 [0.6 ; 0.8] | 1.1 [1 ; 1.2] |
| **OSAS symptoms** | |  |  |  |  |  |
|  | Self-reported daytime sleepiness | 16.2 [8.8 ; 29.6] | 0.1 [0.1 ; 0.1] | 0.4 [0.4 ; 0.5] | 0.2 [0.2 ; 0.2] | 3.7 [2.8 ; 4.8] |
|  | Snoring | 8.2 [3.3 ; 20.2] | 0.1 [0.1 ; 0.1] | 0.5 [0.4 ; 0.7] | 0.2 [0.2 ; 0.3] | 1.8 [1.2 ; 2.8] |
|  | Headache | 7.8 [6.7 ; 9] | 0.2 [0.1 ; 0.2] | 0.3 [0.3 ; 0.3] | 0.4 [0.4 ; 0.5] | 2.4 [2.2 ; 2.7] |
|  | Morning fatigue | 6 [4.5 ; 8] | 0.1 [0.1 ; 0.1] | 0.4 [0.3 ; 0.4] | 0.3 [0.2 ; 0.3] | 2.7 [2.2 ; 3.2] |
|  | Nocturia | 0.7 [0.6 ; 0.8] | 0.3 [0.3 ; 0.4] | 2 [1.8 ; 2.2] | 0.2 [0.1 ; 0.2] | 2.4 [2.1 ; 2.7] |
|  | Near miss accident | 2.5 [2.1 ; 2.9] | 0.2 [0.2 ; 0.3] | 0.4 [0.4 ; 0.5] | 0.6 [0.5 ; 0.8] | 1.7 [1.4 ; 2] |
| **Cardiovascular and metabolic co-morbidities** | |  |  |  |  |  |
|  | Ischemic cardiomyopathy | 0.4 [0.2 ; 0.6] | 2.6 [2 ; 3.2] | 8 [6.4 ; 9.9] | 0.2 [0.1 ; 0.4] | 4.8 [3.8 ; 6] |
|  | Arrhythmias | 0.4 [0.3 ; 0.6] | 2.2 [1.8 ; 2.6] | 6.3 [5.2 ; 7.6] | 0.2 [0.1 ; 0.3] | 3.6 [2.9 ; 4.4] |
|  | Stroke | 0.4 [0.2 ; 0.7] | 1.9 [1.4 ; 2.6] | 4.3 [3.2 ; 5.7] | 0.2 [0.1 ; 0.4] | 2.7 [2 ; 3.6] |
|  | Heart failure | 0.4 [0.2 ; 0.7] | 1.8 [1.3 ; 2.5] | 5.1 [3.8 ; 6.9] | 0.2 [0.1 ; 0.5] | 2.5 [1.8 ; 3.5] |
|  | Diabetes | 0.4 [0.3 ; 0.5] | 1.6 [1.4 ; 1.9] | 9.5 [8.1 ; 11] | 0 [0 ; 0.1] | 5.3 [4.5 ; 6.3] |
|  | Hypertension | 0.3 [0.3 ; 0.4] | 1.8 [1.7 ; 2] | 10.8 [9.6 ; 12.2] | 0.1 [0.1 ; 0.1] | 5.8 [5.2 ; 6.5] |
|  | Dyslipidemia | 0.4 [0.3 ; 0.5] | 0.9 [0.8 ; 1] | 5.3 [4.7 ; 5.8] | 0.1 [0.1 ; 0.2] | 3.7 [3.3 ; 4.1] |
| **Other co-morbidities** | |  |  |  |  |  |
|  | Respiratory co-morbities* | 0.6 [0.4 ; 0.8] | 1.1 [0.9 ; 1.3] | 2.4 [2 ; 2.9] | 0.4 [0.3 ; 0.5] | 2.1 [1.7 ; 2.5] |
|  | Depression | 0.7 [0.6 ; 0.9] | 0.4 [0.4 ; 0.5] | 1.1 [1 ; 1.3] | 0.3 [0.2 ; 0.3] | 1.8 [1.6 ; 2.1] |

* COPD, Asthma and chronic respiratory failure

BMI: body mass index

AHI: apnea hypopnea index

ODI: oxygen desaturation index

Cluster 1: the young symptomatic

Cluster 2: the old obese

Cluster 3: the multi-disease (MD) old obese

Cluster 4: the young snorers

Cluster 5: the drowsy obese

Cluster 6: the MD obese symptomatic
